# Activation of Hair Cell Growth Factors by Linoleic Acid in *Malva verticillata* Seed

**DOI:** 10.3390/molecules26082117

**Published:** 2021-04-07

**Authors:** Hwa Sun Ryu, JiYeon Jeong, Chun Mong Lee, Kwang Sik Lee, Jung-No Lee, Sung-Min Park, Yong-Moon Lee

**Affiliations:** 1 Bio Convergence R&D Center, CoSeedBioPharm Corporation, Heungdeok-gu, Cheongju, Chungbuk 28161, Korea; rnd@coseed.co.kr (H.S.R.); rnd2@coseed.co.kr (J.J.); rnd3@coseed.co.kr (J.-N.L.); rnd4@coseed.co.kr (S.-M.P.); 2 Future Science Research Center, Coreana Cosmetics Corporation, Cheonan, Chungnam 31041, Korea; endless@coreana.co.kr (C.M.L.); kslee@coreana.co.kr (K.S.L.); 3 College of Pharmacy, Chungbuk National University, Cheongju, Chungbuk 28644, Korea

**Keywords:** *Malva verticillata*, linoleic acid, proliferation, growth factor, dihydrotestosterone

## Abstract

Hair loss by excessive stress from work and lifestyle changes has become a growing concern, particularly among young individuals. However, most drugs for alopecia impose a plethora of side effects. We have found the powerful impact of *Malva verticillata* seed extracts on alleviating hair loss. This study further isolated effective chemicals in *M. verticillata* seed extracts by liquid silica gel column chromatography. Under the screening for the growth rate (%) of human follicles dermal papilla cells (HFDPCs), we identified linoleic acid (LA) and oleic acid in *n*-hexane of *M. verticillate* (MH)2 fraction. LA treatment activated Wnt/β-catenin signaling and induced HFDPCs growth by increasing the expression of cell cycle proteins such as cyclin D1 and cyclin-dependent kinase 2. LA treatment also increased several growth factors, such as vascular endothelial growth factor, insulin-like growth factor-1, hepatocyte growth factor, and keratinocyte growth factor, in a dose-dependent manner. Besides, LA significantly inhibited Dickkopf-related protein expression (DKK-1), a primary alopecia signaling by dihydrotestosterone. Our findings suggest that LA treatment may alleviate a testosterone-induced signaling molecule and induces HFDPCs growth by activating Wnt/β-catenin signaling.

## 1. Introduction

Hair loss by excessive stress from work and lifestyle changes has become a growing concern, particularly among young individuals [1]. Indeed, the diverse usage of chemical products leads to increased hair loss and has increased hair transplantation surgery in individuals in their 20s and 30s [2]. Hair loss depends on the rate balance between the hair growth cycle and hair loss [3]. Various factors, such as genetic factors, excessive male hormones, sebum content, and aging, are also involved in abnormal hair loss [4,5].

Hair is growing in hair follicle cells and undergoes a repetitive growth cycle following four stages: anagen, catagen, telogen, and exogen [5,6,7]. The duration of this growth cycle varies depending on the local site and density of hair follicles, and typically it lasts for 2 to 8 years [8]. Generally, the hair growth cycle is repeated 10 to 15 times, while 50–100 hairs are lost per day [9]. Human follicles dermal papilla cells (HFDPCs) and mesenchymal cells located at the bottom of the hair follicle, are the critical cells involved in hair growth cycle regulation [10,11]. At the molecular level, Wnt/β-catenin signaling is an essential factor for maintaining the hair growth activity of HFDPCs [12].

The Food and Drug Administration (FDA)-approved minoxidil or finasteride became known for several side effects [13,14,15,16,17,18]. Therefore, other safer substances have been searched for as alternative medications from natural resources. Of the several herbal candidates, *Malva verticillata* seed has been investigated based on the diverse effects on epithelial cells [19,20]. Recently, we also reported *Malva verticillata* seed activates Wnt/β-catenin proteins [19].

Vegetable oils derived from the seeds of *Malva verticillata* contain polyunsaturated fatty acids, such as linoleic acid (LA) [21]. LA is an essential omega-6 fatty acid for the precursor to arachidonic acid with elongation and saturation [22]. LA is known for its wound-healing and anti-cancer properties [23,24,25]. Besides, LA regulated lymphocyte provides protection and resistance against arteriosclerosis [26,27]. Similarly, conjugated LA, a geometric isomer of LA, exhibited benefits to humans, reducing body fat and, more importantly, suppressing cancer, arteriosclerosis, and diabetes [28,29,30,31]. A short supplement of fatty acids, including LA, is related to hair loss, although its mechanism is unclear.

In the present study, we characterized the bioactive lipids in the seeds of *Malva verticillata* by several steps of liquid extraction and collection via silica gel column chromatography. Purified lipids were LA and oleic acid, which give proliferative effects on HFDPCs via activating Wnt/β-catenin proteins.

## 2. Results

### 2.1. Malva verticillata Seed Extraction and Active Ingredient Separation

**Figure 1:**
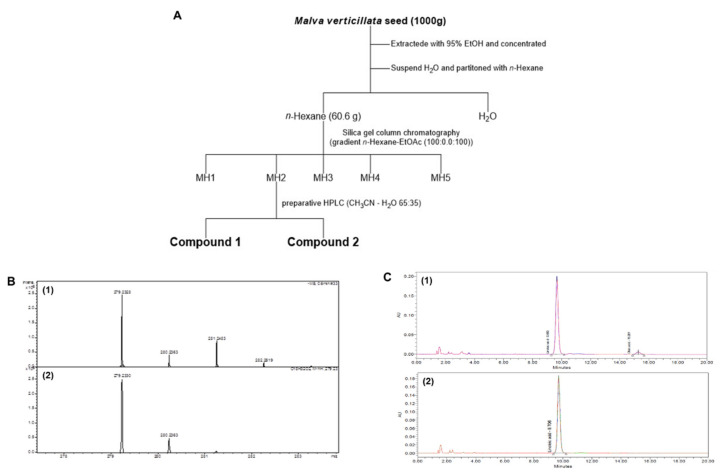
The scheme (**A**) of isolated compounds from *Malva verticillata* seed and LC-mass data (**B**); B-1 shows mass spectroscopy of Compound **1** [M − H]^−^ 279.23, and B-2 shows the mass spectroscopy of Linoleic acid standard (Sigma-Aldrich, St. Louis, MO, USA). And HPLC data of LA (**C**); C-1 shows HPLC chromatogram of Compound **1** T_R_ 9.7 min and C-2 shows HPLC chromatogram of Linoleic acid standard (Sigma-Aldrich, St. Louis, MO, USA).

We purified compounds **1** and **2** using preparative high-performance liquid chromatography (CH_3_OH:H_2_O = 70:30) for the *n*-hexane of *M. verticillate* (MH)2 fraction among the five fractions (MH1–5) fractionated using silica gel column chromatography on the n-hexane part of dried *Malva verticillata* seeds (Figure 1). We structurally identified the two separated compounds were by comparing the reported spectroscopic data [32,33] and the spectrum data obtained through nuclear magnetic resonance, mass spectroscopy, and HPLC analysis. First, we confirmed the structures by NMR (data not shown). Then we identified LA’s structure by comparing mass spectroscopy (Figure 1B) and HPLC chromatogram (Figure 1C). The retention times of compounds **1** and **2** were finally identical to LA and oleic acid on HPLC chromatograms to re-confirm the compounds by co-eluting with the fatty acid standard. Compounds **1** and **2** were identified as LA and oleic acid, respectively.

### 2.2. Effects of Malva verticillata Extract and LA Treatment on Hair DPC Proliferation

**Figure 2:**
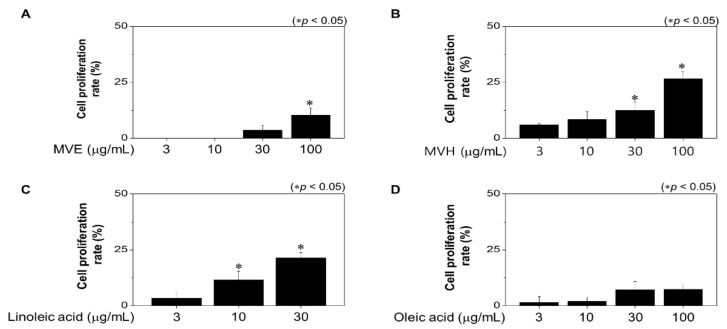
Effect on hair cell proliferation. Human follicles dermal papilla cells (HFDPCs) were treated with *M. verticillate* seed EtOH extract (MVE) (**A**), *n*-hexane fraction (MVH) (**B**), linoleic acid (**C**), and oleic acid (**D**) for 48 h. We measured cell proliferation by MTT at 550 nm. All data were as mean ±SEM of three separate experiments performed in triplicate (* *p* < 0.05).

We next assessed the inhibitory effects of treatment with extracted *Malva verticillate* and the *n*-hex fraction on the proliferation of HFDPCs. *Malva verticillata* seed extract (MVE) treatment significantly increased cell proliferation by 10.42% at 100 µg/mL concentration (*p* < 0.5, Figure 2A), whereas the n-hexane fraction (MVH) showed significant effects at 30 µg/mL concentration and increased cell proliferation by up to 26.62% at 100 µg/mL (*p* < 0.5, Figure 2B). Furthermore, LA treatment at 10 µg/mL showed a significant increase in cell proliferation rate, which was increased by up to 21.46% at 30 µg/mL concentration (*p* < 0.5, Figure 2C). Additionally, there was no significant effect in response to 100 ug/mL of oleic acid (Figure 2D). These findings suggest that LA mediates *Malva verticillata* seed extract’s efficacy in increasing hair cell population.

### 2.3. Effects of LA Treatment on the Wnt/β-Catenin Pathway

**Figure 3:**
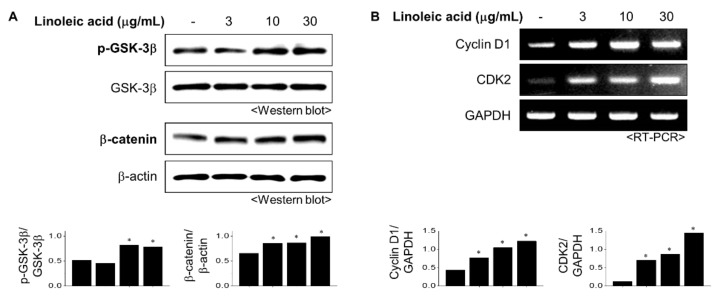
Effect of LA on Wnt/β-catenin signaling and cell cycle. (**A**) LA treated into HFDPC cells for 6 h. Total cell extracts were blotted with GSK-3β and β-catenin antibodies (**B**). The mRNA levels of cyclin D1 and CDK2 were measured using reverse transcription-polymerase chain reaction (RT-PCR). Band intensities were quantified using ImageJ 1.47 software and normalized to β-actin (**A**) or GAPDH (**B**). All data showed mean ±SEM of three separate experiments performed in triplicate (* *p* < 0.05).

The Wnt/β-catenin pathway regulates various physiological phenomena in cells and plays a vital role in regulating the proliferation of dermal papilla cells (DPCs) [12]. Assessment of the effects of LA treatment on the Wnt/β-catenin pathway revealed a significant increase in the phosphorylation of GSK-3 at 10 µg/mL concentration (Figure 3A). LA treatment also sequentially increased β-catenin expression in the cytoplasm in a concentration-dependent manner (Figure 3A). Thus, we inferred that LA might induce cell proliferation by activating the Wnt signaling pathway.

Activation of the Wnt/β-catenin pathway leads to various genes involved in the cell cycle, proliferation, and survival. The effect of LA treatment on the cell cycle was evaluated using RT-PCR. The cyclin–cyclin-dependent kinase (CDK) protein complexes controlled the cell cycle. LA treatment increased the expression levels of cyclin D1 and CDK2, the two essential proteins involved in regulating the cell cycle, in a concentration-dependent manner (Figure 3B). These results suggest that LA regulates the proliferation of DPCs through modulation of the cell cycle.

### 2.4. Effects of LA Treatment on Expression of Hair Growth Factors

**Figure 4:**
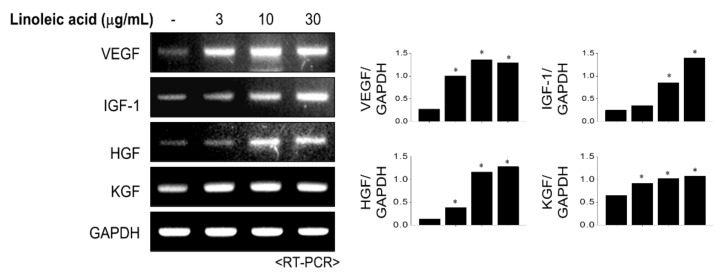
The effect of linoleic acid (LA) on growth factor expressions. LA treated into HFDPC cells with various concentrations for 6 h. The mRNA levels of growth factors were measured using reverse transcription-polymerase chain reaction (RT-PCR). Band intensities were quantified using ImageJ 1.47 software and normalized to GAPDH. All data showed mean ±SEM of three separate experiments performed in triplicate (* *p* < 0.05).

Growth factors such as vascular endothelial growth factor (VEGF), insulin-like growth factor (IGF), and fibroblast growth factor are involved in the growth and differentiation of hair papilla cells and regulate new hair formation. Fibroblast growth factor and IGF-1 promote hair growth by inducing follicle tissue growth and hair follicle cell proliferation. VEGF activates hair growth by causing blood vessel formation to provide nutrients to hair follicle cells. RT-PCR was performed to evaluate the effect of LA treatment on hair growth factors (Figure 4). The primer of RT-PCR was shown in Table 1. LA significantly increased the gene expression level of VEGF. Furthermore, LA also increased the expressions of IGF-1, hepatocyte growth factor (HGF), and keratinocyte growth factor (KGF) in a dose-dependent manner. These findings suggest that LA treatment promotes hair growth by inducing the expression of growth factors.

### 2.5. Inhibitory Effects of LA Treatment on Dihydrotestosterone-Induced DKK-1 Expression

**Figure 5:**
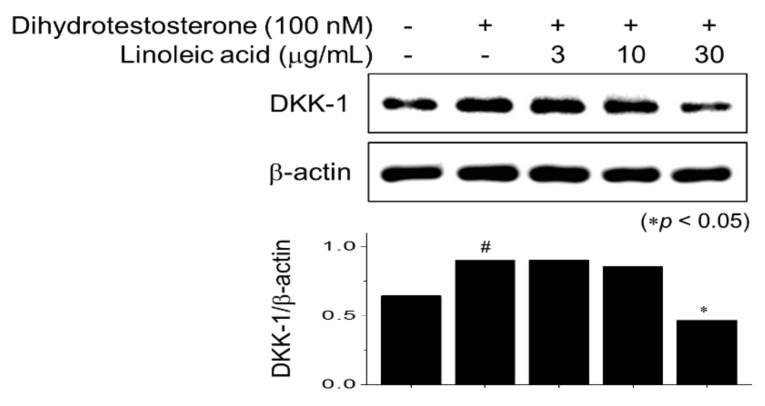
LA suppressed the dihydrotestosterone (DHT)-induced Dickkopf-related protein expression (DKK-1) expression. HFDPC cells were stimulated with DHT for 2 h and treated with various LA concentrations for 6 h. Total cell extracts were blotted with DKK-1 and β-actin antibodies. Band intensities were quantified using ImageJ 1.47 software. Significance was determined versus DHT-untreated (^#^ *p* < 0.05) or DHT only-treated cells (* *p* < 0.05). All data showed mean ±SEM of three separate experiments performed in triplicate.

Another major factor that causes hair loss occurs when testosterone, a male hormone, is converted into dihydrotestosterone by 5α-reductase. When dihydrotestosterone binds to the androgen receptor, Dickkopf-related protein (DKK)-1 expression induces hair cell death, and inhibition of Wnt/β-catenin signaling leads to hair loss. LA treatment significantly inhibited the expression of DKK1 increased by dihydrotestosterone treatment at a concentration of 30 µg/mL (Figure 5). DKK-1 protein encoded by the DKK1 gene antagonizes the Wnt/β-catenin signaling by inhibiting the Wnt coreceptors [34]. Thus, we concluded that LA treatment might also be effective against testosterone-induced hair loss.

## 3. Discussion

Hair loss is explained by the imbalance between the difference in hair growth and hair falling out. Hair loss, which was traditionally a concern for the elderly, is now also often observed in younger age groups due to excessive stress, changes in dietary habits, and environmental factors. The most important mechanism underlying abnormal hair loss is dysregulation of the proliferation of DPCs that produce hair. DPCs are special mesenchymal cells that play a crucial role in hair growth. They exist in hair follicles and are supplied with nutrients by capillaries to activate hair matrix cells to promote hair growth. DPCs also regulate hair follicle development by secreting cytokines and growth factors or transmitting various signals through direct interaction. Mouse model studies suggested that the number of DPCs control the size and shape of the hair.

Moreover, hair follicles could not generate new hair if the number of DPCs were below a specific threshold value. A growing interest in treating hair loss has led to the quest for identifying alternative, safe, and natural therapeutic sources. To this end, in the present study, we investigated the efficacy of treatment with the bioactive components of *Malva verticillata*, particularly LA, in preventing hair loss.

Here, we showed that treatment with LA extracted from *Malva verticillata* seeds significantly increased the proliferation of DPCs, suggesting that it could contribute to hair regeneration [11]. Moreover, LA treatment could induce activation of Wnt/β-catenin signaling. In the Wnt/β-catenin signaling pathway, the absence of external signals leads to phosphorylation of β-catenin by GSK-3, and β-catenin is then ubiquitinated and degraded. However, when the Wnt ligand binds to its receptor, the activity of GSK-3 is suppressed, inhibiting β-catenin degradation, following which β-catenin moves into the nucleus. Subsequently, β-catenin can regulate the expression of genes such as those encoding cyclin D and *c-Myc*, which induces hair cell proliferation [35]. The treatment with mixed herbal extracts of avocado, marshmallow, chamomile, thyme, rosemary, and sedge nettle increased cyclin D1 expression CDK4 in DPCs [36]. LA treatment also induces cell proliferation by activating the cell cycle through increased expression of cyclin D1 and CDK2, which leads to activation of the Wnt/β-catenin pathway.

**Figure 6:**
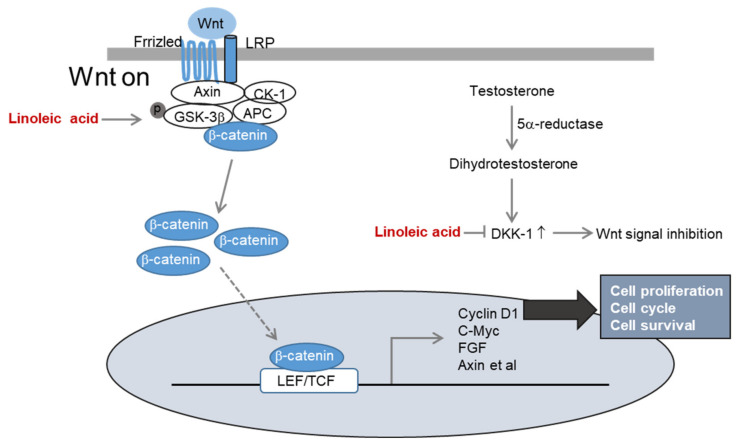
The mechanism of linoleic acid in hair loss improvement.

Another mechanism of hair loss caused by hormonal imbalance occurs when dihydrotestosterone, converted from testosterone by 5α-reductase, is produced excessively. When dihydrotestosterone binds to DPC, the expression of DKK-1, which induces apoptosis, leading to the death of hair matrix cells, is a specific hypothesis on hair loss. In a co-culture experiment of DPCs and outer root sheath (ORS) keratinocytes, the expression of DKK-1 was increased by dihydrotestosterone, reducing the proliferation rate of ORS keratinocytes [37]. Another vital aspect of dihydrotestosterone-induced hair loss is reduced cell proliferation via inhibition of Wnt/β-catenin signaling. LA activates Wnt/β-catenin signaling and effectively inhibits the expression of DKK1 by dihydrotestosterone (Figure 6).

## 4. Materials and Methods

### 4.1. Materials

Dimethyl sulfoxide (DMSO), 3-(4,5-dimethylthiazol-2-yl)-2,5-diphenyltetrazolium bromide (MTT), and β-actin (#A5316) were from Sigma-Aldrich (St.Louis, MO, USA). DKK-1 (#SC-374574) antibody were from Santa Cruz Biotechnology (Santa Cruz, CA, USA) and GSK-3β (#9315S), p-GSK-3β(#9323S), and β-catenin(#9562S) antibodies were from Cell Signaling Technology (Danvers, MA, USA). Phosphate-buffered saline (PBS) and penicillin/streptomycin were from Gibco (Grand Island, NY, USA).

### 4.2. Malva verticillata Seed Extraction and Active Ingredient Separation

Dried *Malva verticillata* seeds (1 kg) were extracted twice at room temperature using 95% ethanol (10 L). We concentrated the ethanol extract using a rotary evaporator (Basis Hei-VAP, Heidolph, Germany) and a vacuum pump (Rotavac valve control, Heidelberg, Germany). The concentrated product (408 g) was then suspended in 20% ethanol (5 L), and the product was fractioned according to the order of solvent polarity to obtain *n*-hexane (hex), dichloromethane, ethyl acetate, n-butanol, and water fractions. Among the fractions obtained, the n-hex fraction (60.6 g) was subjected to silica gel column chromatography, and a total of five portions (MH1–5) were obtained using *n*-hex/EtOAc (100:0–0:100). Subsequent purification of the MH2 fraction using preparative high-performance liquid chromatography (CH_3_OH:H_2_O = 70:30) led to the identification of compounds 1 and 2.

### 4.3. Cell Culture

Human hair follicle DPCs were purchased from Promo Cell (Heidelberg, Germany). We used the DPC-specified growth medium for the DPC experiments. The cells were passaged every 3–4 days and incubated at 37 °C and 5% CO_2_.

### 4.4. Cell Proliferation Assay

HFDPCs were seeded at 2 × 10^4^ cells/well in a 96-well plate and incubated for one day at 37 °C and 5% CO_2_. The cells were treated with the *Malva verticillata* seed extracts and LA at various concentrations and incubated for 48 h. Subsequently, 20 µL MTT (5 mg/mL) reagent was added to each well, and the cells were incubated again for 3 h. After removing the supernatant, we subtracted 100 µL of DMSO to dissolve the formazan completely. Absorbance at a 550-nm wavelength was measured using a microplate reader (SpectraMax i3x, Molecular Devices, San Jose, CA, USA).

### 4.5. Reverse Transcription Polymerase Chain Reaction (RT-PCR)

Total RNA from HDFPCs was extracted using TRIzol reagent, and cDNA was synthesized from 2 µg of the total RNA [31]. We used PCR premix (Bioneer, Daejeon, Korea); the primers’ sequence in Table 1. The PCR products were analyzed using 2% agarose gel stained with eco dye, and the product band intensities were quantified using ImageJ 1.47 software (NIH, Bethesda, MD, USA).

### 4.6. Western Blotting (WB)

HFDPCs were seeded at 2 × 10^5^ cells/mL in a 6-well plate in 2 mL of medium and incubated for 24 h. The cells were treated with various concentrations of LA for 48 h. Next, they were washed once with 1× phosphate-buffered saline (PBS) and lysed using cell lysis buffer (#9803, Cell Signaling Technology, Danvers, MA, USA) with protease inhibitors (#5872, Cell Signaling Technology, Danvers, MA, USA). After centrifugation at 12,000 rpm at 4 °C for 15 min, the supernatant was separated and used as a protein solution. The protein was quantified using a bicinchoninic acid assay (Thermo Scientific, MA, USA). The electrophoretic separation using 10% sodium dodecyl sulfate–polyacrylamide gel electrophoresis at 95 V for 2 h. The separated bands were then transferred to a polyvinylidene fluoride membrane and blocked for 1 h using 5% skim milk. The primary antibody dissolved in 5% bovine serum albumin was added to the membrane and reacted at 4 °C for 24 h. Next, a secondary antibody was added and reacted for 2 h. A super signal chemiluminescent substrate (Thermo Scientific, MA, USA) was used to identify the protein signals quantified using ImageJ 1.47 software.

### 4.7. Statistical Analysis

All experiments were repeated at least three times, and the results are expressed as mean ± SEM. Data were analyzed using one-way ANOVA (*p < 0.05).

## 5. Conclusions

In summary, LA isolated from *Malva verticillata* seeds activated Wnt/β-catenin signaling to promote the cell cycle and growth factor secretion, inducing proliferation of DPCs and hair growth. Moreover, it alleviated DKK-1 expression associated with testosterone, which is another cause of hair loss. This study presents a potential alternative and effective natural therapeutic agent against hair loss. The present results suggest a molecular basis for LA’s anti-hair loss effect in hair dermal papilla cells, but clinical studies will be needed to ensure LA’s outcomes. Besides, it will be required to test the direct impact of LA on the Wnt pathway activation.

## Figures and Tables

**Table 1:** Polymerase chain reaction (PCR) primer sequences.

Name	Primer		Gene Bank Accession
	Forward	Reverse	
Cyclin D1	CGTGGCCTCTAAGATGAAGG	TGCGGATGATCTGTTTGTTC	CR542099.1
CDK2	AAAGCCAGAAACAAGTTGACG	GAGATCTCTCGGATGGCAGT	X62071.1
VEGF	AAGGAGGAGGGCAGAATCAT	TTTCTTGCGCTTTCGTTTTT	NM001025368
IGF-1	TCAACAAGCCCACAGGGTAT	CGTGCAGAGCAAAGGAT	A29119.1
HGF	GCCTGAAAGATATCCCGACA	TTCCATGTTCTTGTCCCACA	NM001010932.3
KGF	GACATGGATCCTGCCAACTT	AATTCCAACTGCCACTGTCC	S81661.1
GAPDH	TCCATGACAACTTTGGTATC	TGTAGCCAAATTCGTTGTCA	NM001289745.2

## Sample Availability

Samples of *Malva verticillata* seed extracts are available from the authors.

## Abbreviations

CDK	Cyclin-dependent kinase
DPC	Dermal papilla cells
LA	Linoleic acid
HFDPC	Human hair dermal papilla cells
DKK-1	Dickkopf-related protein-1
